# Relationship between human resources strategies and organizational performance based on the balanced scorecard in a public hospital in Iran: a cross-sectional study

**DOI:** 10.1186/s12913-022-07767-z

**Published:** 2022-03-18

**Authors:** Ebrahim Nafari, Behrooz Rezaei

**Affiliations:** 1 Department of Health & Health Services Management, Medical Faculty, Shahrekord Branch, Islamic Azad University, Shahrekord, Iran; 2 Nursing & Midwifery Faculty, Falavarjan Branch, Islamic Azad University, Isfahan, Iran

**Keywords:** Human resources strategies, Human resources, Organizational performance, Balanced scorecard, Hospital, Hospital Staff, Iran

## Abstract

**Background:**

Today, due to the complex environment and rapidly changing of health industry, hospitals should optimize their organizational performance to achieve a competitive advantage. One of the important factors for achieving a competitive advantage is effective human resource management through the application of appropriate human resources strategies. This study investigated the relationship between human resources strategies and organizational performance based on balanced scorecard.

**Methods:**

A cross-sectional study was conducted among clinical and administrative staff in a public hospital in Isfahan province, Iran. All eligible staff entered the study (*n*=200). Data were collected using two self-reported questionnaires during July 2018. The main questionnaire contained 32 items that investigated human resources strategies (including seven types of strategies) and organizational performance based on the balanced scorecard approach. Data were analyzed via Pearson correlation coefficient and multivariate regression using SPSS/21 software (*P*<0.05).

**Results:**

In this study, 154 questionnaires were completed and returned (return rate=77%). Human resources strategies and organizational performance were evaluated at the intermediate level. There was a significant positive correlation between human resources strategies and organizational performance (*r*=0.73, *p*=0.001). The organizational performance in learning & growth perspective had the highest correlation (*r*=0.669, *p*=0.001) with human resources strategies while the financial perspective had the lowest (*r*=0.455, *p*=0.001). Multivariate regression analysis showed that all human resources strategies were effective on organizational performance (*R*=0.998, *R*2=0.997, ADJ.*R*2=0.997). Staff training and development strategy (Beta=0. 265, *p*=0.000), staff compensation & reward strategy (Beta =0.212, *p*= 0.000) and recruitment strategy (Beta = 0.208, B, *P*=0.000) had the greatest impact on organizational performance, respectively.

**Conclusions:**

To improve hospital performance (especially in non financial perspectives), the development of human resources strategies (especially staff training and development strategy, staff compensation & reward strategy, and compensation strategy) can be considered by hospital managers. The findings of this study can be used for developing hospital performance in a similar context.

## Background & introduction

Today, human resources are recognized as resources that can create a valuable competitive advantage for an organization [1]. The competitive advantage can be achieved through employees and various human resources management activities [2]. However, to achieve a competitive advantage, organizations must use tools to attract, retain, and motivate their human resources, which are called Human Resources Strategies (HRS) [1, 3]. Otherwise, they will lose the ability to adapt to today's complex environment and the ability to use human resources effectively [3]. Success in human resources management requires the development and implementation of appropriate HRS [4].

Strategic human resources management is considered a link between HRS and the business strategy of the organization [5]. The goal of strategic human resources management is how organizations can use their human resources to improve their competitive performance [6].

Human resources strategies are the decision-making model of human resources system policies in the field of business strategy and competitive context [5]. These strategies refer to the overall understanding of the organization on how to manage different aspects of employees to simultaneously strengthen the competitiveness and well-being of employees. Human resources strategies are the common view of management and employees about the type of human resources policies that should be implemented in the organization [4]. These strategies reflect what the organization intends to do about the various aspects of its human resources management policies and practices [7]. Successful HRS determine what factors affect the success of an organization [4]. Continuous development of human resources is a key factor in achieving a sustainable competitive advantage for any organization [5].

Understanding the nature of the HRS is impossible without considering intra-organizational policies and environmental/institutional forecasts. Strategic human resources management is a process through which organizations seek to link the human and social capital of their members with the strategic needs of the organization, while HRS is a roadmap to provide this link and emerging human resources which organizational leaders use [5].

However, human resources management decisions are to have a significant and unique impact on organizational performance (OP) [8]. With applying HRS, managers can achieve optimal OP by making appropriate use of opportunities and threats [5].

Performance refers to both behaviors and outcomes. This means that in evaluating performance, both employees' behaviors and their results should be considered [7]. Evaluating the OP improves the quality of products and services and helps to reduce costs [9]. Improving the OP requires having a suitable model for performance evaluation and the balanced scorecard (BSC) can be used as a successful model to evaluate the performance of service organizations, especially hospitals [10].

In the 1990s, Kaplan and Norton recognized the needs and requirements of new organizations and introduced the Balanced Scorecard (BSC) as a performance improvement model. This model is known as a comprehensive framework for performance appraisal and strategy development. The BSC establishes a relationship between short-term and long-term goals, financial and non-financial metrics, internal and external performance, internal and external stakeholders of the organization. This model is one of the most successful organizational performance management systems [11].

The BSC is a combination of criteria for evaluating the past, present and future performance of the organization, which evaluates non-financial indicators along with financial criteria. This model manages the implementation of strategies and measures the performance of the organization in four perspectives; financial, customer, internal business process and learning & growth. The BSC reflects the vision and mission of the organization in these four perspectives [12, 13].

The financial perspective identifies the financial needs and performance of the organization and considers criteria for financial measurement. Financial metrics show what financial results have ultimately been achieved by successfully achieving the goals in the other three perspectives. By the customer perspective, the level of customer satisfaction is assessed through quantitative and qualitative metrics in relation to services and products. This aspect includes several main and sub-indicators. The main indicators are: customer satisfaction, customer retention, new customer attraction, customer profitability and market share. In the internal business process perspective, the processes that lead to value creation for customers and shareholders are identified. This aspect emphasizes the internal business results that lead to financial success and meet customer expectations. The learning & growth perspective focuses on the way employees are trained, acquired and applied knowledge to maintain a competitive advantage [12, 14].

After determining the goals and criterias in the customer perspective and the internal business process perspective, the skills and capabilities needed by employees to achieve these goals and criterias are identified and indicators are provided to control the progress of these skills and knowledge [14, 15].

Organizations should be able to design and implement organizational strategies in the form of measurable performance indicators. The BSC is a set of measurable indicators derived from organizational strategies [14, 15]. However, after more than twenty years of research and application, this approach has emerged in some studies in healthcare organizations [15].

Over the past decades, the focus of research on HRS has been in three areas. The first area is related to the formulation and implementation of these strategies. In which not only how to develop the most effective HRS, but also the organizational characteristics of adopting specific methods of these strategies have been considered. In the second area, the content of HRS (including dimensions and variables), especially the policies and different methods of these have been considered. While in the third area, the most attention of researchers has been focused on the effects of HRS, especially on OP [5].

Empirical evidence convincingly demonstrates the positive effect of strategic human resource management on organizational performance in terms of increased productivity, higher profitability, and lower employee turnover rate. While the public sector is the largest provider of community services, these research have relied more on evidence from private sector organizations. In many countries, the public sector plays a major role in ensuring public health and well-being. Nevertheless, the public sector organizations are facing declining resources and increasing demand for accountability and improving the service quality. All these issues have conducted the study on human resources and performance of public service organizations an important context of research [16].

Numerous studies have been conducted in industrial organizations. Research conducted in healthcare institutions indicates that the successful implementation of HRS improves OP and hospital services [2, 3, 6, 17–21]. In this regard, a study in sixteen Jordanian public hospitals showed that strategic human resources management factors had a significant relationship with positive hospital performance [6]. Michael et al. (2006) conducted a study in fifty two UK hospitals and found that HRS has a significant relationship with reducing patient mortality [20]. Additionally, Vermeeren et al. (2014) conducted a study in 162 Dutch healthcare institutions and showed that human resources management activities affect financial, organizational, and staff results [2].

Hospitals as an important part of service organizations are changing and complicating faster than other organizations. Modern health care is changing and becoming more complex due to new patterns of diseases, advanced technologies, unpredictable patient needs, and diverse personnel needs [22]. To survive in such conditions, evaluating the performance of the organization can play an effective role in maintaining the quality of hospital services [23]. Performance measurement is a key factor for any organization in order to control the implementation of the organizational strategies [24]. So development of research in this area must be more considered.

One of the main goals of human resources strategies is to increase performance and organizational productivity. However, issues such as huge investments, the use of expensive medical technology, and the constant increase of hospital costs have made it even more necessary to optimize organizational performance of hospitals. Although past research indicates that the successful implementation of HRS improves hospital performance, but the available national information is insufficient in Iranian context. As far as the authors know, a national study has not previously been conducted to investigate the relationship between HRS and OP based on the balanced scored card approach in Iranian hospitals. Therefore, this study aimed to investigate the relationship between HRS and OP based on the balanced scorecard in a public hospital in Isfahan province, Iran.

## Methods

### Study design, setting & sample

A cross-sectional study was conducted in a public hospital in Isfahan, Iran. All eligible staff of the hospital entered the study. Data were collected using two Persian questionnaires in July 2018. The statistical population consisted of all clinical and administrative staff of the hospital (*N* = 250). The staff with full-time employment, work experience higher than one year, and associate education level or higher who informed consent to participate, were included in the study (*n*=200). Incomplete questionnaires were excluded from the study. Finally, the data of 154 participants were analyzed.

### Data collection tools

The instrument of this study consisted of the two Persian questionnaires, which printed versions of those were completed by the participants via a self-report method. The first questionnaire was a demographic questionnaire that included personal information including age, gender, level of education, work experience, and type of employment. The second questionnaire consisted of 32 items on a 5-point Likert scale (from very low=1 to very high= 5), which simultaneously assessed employees' perspectives on HRS and OP based on BSC. The items related to HRS taken from the past research in seven dimensions include; performance management (4 items), staff training and development (8 items), staff compensation and reward (4 items), recruitment (4 items), equal opportunities (4 items), staff relations (4 items), and flexible work programs (4 items). Organizational performance items are designed based on a balanced scorecard, which is derived from the view of Kaplan & Norton (1996) in four perspectives including; the customer perspective (8 items), financial perspective (8 items), internal business process perspective (8 items), and learning and growth perspective (8 items) [13, 25–27]. The classification of questions in this questionnaire is presented based on the studied variables in Table 1. For example, by questions 13-16, the effect of recruitment strategy on OP based on BSC in four perspectives; finance (question 13), customer (question 14), internal business process (question 15) and learning and growth (question 16) were measured.

**Table 1:** The classification of questions in the questionnaire based on the studied variables

Variables	Categories	N
Human Resources Strategies		
	Performance Management Staff training and development Staff compensation and reward Recruitment Equal opportunities Staff relations Flexible work programs	1-4 5-8, 17-20 9-12 13-16 21-24 25-28 29-32
Perspectives of OP based on BSC		
	Financial Perspective Customer Perspective Internal Business Process Perspective Learning & Growth Perspective	1,5,9,13,17,21,25,29 2,6,10,14,18,22,26,30 3,7,11,15,19,23,27,31 4,8,12,16,20,24,28,32
Total items	-	32

Ghasemi (2013) designed this questionnaire and confirmed its validity using the content validity method. Its reliability was also confirmed in the study by Ghasemi (2013) using Cronbach's alpha coefficient (α=0.88) [9]. In our study, the reliability of the questionnaire was confirmed using Cronbach's alpha coefficient (α=0.94).

### Data analysis

Data were analyzed using descriptive statistics (including frequency distribution tables, mean and standard deviation) and analytical statistics (including Pearson correlation coefficient & multivariate regression analysis) by SPSS 21 software at significance level of 0.05. The Kolmogorov-Smirnov test was used to evaluate the normality of the data.

## Results

In this study, 154 questionnaires were completed and returned (return rate = 77%). Descriptive results showed that most participants (75.5%) were women and half of them (49.4%) were in the age group of 30-40 years. More than half of the participants (58.4%) had a bachelor's degree. Also, 43.5% of the participants were nurses working in inpatient wards. Nearly half of the participants had more than 10 years of work experience and their type of employment been permanent (Table 2).

**Table 2:** Demographic information of the participants working in a public hospital

Variables	Categories	N (%)
Gender	Male Female	116 (75.5) 33 (24.4)
Education level	Associate's degree Bachelor’s degree Master’s degree or higher	45 (29.2) 90 (58.4) 19 (12.3)
Age (years)	<30 30–40 40–50 >50	36 (23.40) 76 (49.4) 35 (22.7) 7 (4.5)
Work experience (years)	<5 5–10 10–15 >15	49 (31.8) 29 (18.8) 32 (20.8) 44 (28.6)
Job category	Administrative Financial Medical Nursing Other	37 (24.0) 8 (5.2) 12 (7.8) 67 (43.5) 30 (19.5)
Employment type	Permanent Contract Temporary	69 (44.80) 71 (46.1) 14 (9.1)
Total	**-**	**154 (100)**

According to the score ranking of the questionnaire, the results showed that the mean score of both HRS and OP were at the intermediate level. However, among the dimensions of the HRS, staff training and development, recruitment, and staff compensation and reward scored the highest while, among the various perspectives of OP, the internal business process perspective received the highest score (Table 3).

**Table 3:** Descriptive statistics indicators of HRS and OP based on the participants viewpoints (according to Likert scale 1-5)

Variable	Dimensions	N (Items)	Mean (SD)	Max (Min)	Level/rank
HSR	Performance management	4	3.31 (0.45)	4.13 (2.40)	Medium Level
	Staff Training and development	8	3.32 (0.43)	4.22 (2.49)	Medium Level
	Staff Compensation & Reward	4	3.45 (0.44)	4.13 (2.67)	Medium Level
	Recruitment	4	3.50 (0.69)	6.33 (2.40)	Medium Level
	Equal opportunities	4	3.07 (0.55)	3.84 (1.95)	Medium Level
	Staff relations	4	3.20 (0.44)	4.06 (2.24)	Medium Level
	Flexible work programs	4	3.98 (0.69)	4.00 (2.60)	Medium Level
	The total score of HSR	32	3.36 (0.45)	4.39 (2.39)	Medium Level
OP	Customer Perspective	8	3.26 (0.64)	4.67 (2.00)	Medium Level
	Financial Perspective	8	3.20 (0.52)	4.00 (2.01)	Medium Level
	Internal business process Perspective	8	3.48 (0.60)	5.00 (2.02)	Medium Level
	Learning and growth Perspective	8	3.22 (0.61)	4.00 (2.00)	Medium Level
	The total score of OP	32	3.28 (0.58)	4.41 (2.00)	Medium Level

*HRS*: Human Resources Strategies, *OP:* Organizational Performance

Kolmogorov-Smirnov test showed that the main variables of the study including the scores of HRS and OP had normal distribution (*p*> 0.05).

The results of the Pearson correlation coefficient test showed that HRS positively correlated with all perspectives of OP. In addition, Human Resources Strategies had the highest correlation with OP in the learning and growth perspective (*r*=0.669), while these strategies had the lowest correlation with OP in the internal business process perspective (*r*=0.359) (Table 4).

**Table 4:** Pearson correlation coefficient between HRS and perspectives of OP

Item	1	2	3	4	5	6
1. Customer Perspective	1					
2. Financial Perspective	0.870	1				
3. Internal Business process Perspective	0.925	0.870	1			
4. Learning and growth Perspective	0.913	0.832	0.895	1		
5. OP (total score)	0.950	0.941	0.920	0.964	1	
6. HSR (total score)	0.455^a^	0.455^a^	0.359^b^	0.669^a^	0.730^a^	1

*OP:* Organization Performance, *HRS:* Human Recourses Strategies

^a^Correlation is significant at the 0.001 level (2-tailed)

^b^Correlation is significant at the 0.01 level (2-tailed)

Multivariate regression analysis was used to simultaneously investigate the effect of seven dimensions of HRS on OP. Before preparing the regression model and confirming the validity of the regression model, the multivariate regression hypotheses were examined and confirmed firstly. The results of regression analysis showed that all types of HRS were effective on OP (*R*=0.998, *R*2=0.997, ADJ.R2=0.997). Among the dimensions of HSR, staff training and development strategy (Beta=0. 265, *p*=0.000), staff compensation and reward (Beta=0.212, *p*=0.000) and recruitment strategy (Beta=0.208, B, *P*=0.000) had the greatest impact on OP, respectively. However, staff relations strategy had the lowest impact on OP (Beta=0.156, *p*=0.000) (Table 5).

**Table 5:** Multivariate regression results in input style to predict the OP through various dimensions of the HRS

Predictor variables	B	S.E	Beta	T	P
Constant	0.860	0.407	-	2.112	0.037
Staff training and development strategy	1.204	0.034	0.265	35.407	0.000*
Staff compensation strategy	0.973	0.029	0.212	33.022	0.000*
Recruitment strategy	1.101	0.037	0.208	29.579	0.000*
Equal opportunities strategy	1.008	0.031	0.207	35.520	0.000*
Staff relations strategy	0.917	0.051	0.156	17.879	0.000*
Flexible work programs strategy	1.069	0.044	0.191	24.064	0.000*
Performance management strategy	1.074	0.040	0.179	27.092	0.000*
**Model Summary**	*R*=0.998	*R*^2^=0.997	ADJ.R^2^=0.997	S.E=1.171	Durbin-Watson=1.902

* Significant at the 0.001 level (2-tailed)

## Discussion

Human Recourses Strategies are known as the common view of management and staff on the type of human resources policies applicable in an organization. These strategies are the important task of human resources management and should be designed based on continuous dialog with stakeholders and employees of the organization. The successful HRS determine what influence the success of an organization, what values the organization respects, and what actions the organization take when using and strengthening its critical resources [4].

The present study showed that HRS was at an intermediate level from the viewpoint of the hospital staff. This finding is consistent with the research by Ashourpour et al. (2015) in psychiatric hospitals in Tehran [17]. Each human resource strategy must have two key elements: strategic goals (that is, what the strategy is supposed to achieve), and an action plan (ie, tools), through which the desired goals are achieved. Human Resources strategies focus on the organization's goals of what needs to be done and what needs to be changed [1]. These strategies are joint development program of human resources and management that include a long-term plan to determine the policies, goals and actions of employees [4]. HSR indicate the capacity of the organization and a mechanism to improve staff efficiency. These strategies are the basis of the organization's human resource management and a prerequisite for the success of human resource management. Therefore, it seems necessary for hospital management to improve staff perceptions of HSR through a practical program.

The present study showed that the OP based on the balanced scorecard was at the intermediate level. In the meantime, performance scored the highest score in terms of internal business process and lowest score in terms of financial from the perspective of hospital staff. In the study by Aghili and Tutunchi (2015), the performance of the hospital in internal business process perspective got the highest score, while in the study by Jahanyan et al. (2017) the performance in the financial perspective acquired the highest score [10, 23]. The balanced scorecard emphasizes tight linkage of measurement to strategy. The linkage elevates the role for nonfinancial measures from an operational checklist to a comprehensive system for strategy implementation [25]. Although the OP reported in the present study is at intermediate level, it seems, the safety and optimal quality of hospital services require high organizational performance.

In line with the main purpose of this study, linear regression analysis showed that all of the HRS were effective on the OP. In the meantime, staff training and development strategy, staff compensation and reward strategy, and recruitment strategy were the strongest predictors of the OP, while staff relations strategy had the lowest impact on OP. Most national and international research points out that successful implementation of HRS improve the performance of hospitals [2, 3, 6, 17–21].

In this regard, Ashourpour and Najafi (2015), and Ilyasi et al. (2016) showed that recruitment strategy had the most effect on hospital staff productivity while the training & development strategy had the least effect [17, 18]. In addition, Nasiri et al. (2013) found that staff training and development strategy, staff relations strategy, and performance management strategy correlated with OP significantly while recruitment strategy and staff compensation and reward strategy had not significant effect on hospital performance [3]. However, Hameed & Mohamed (2016) found that only the recruitment strategy and the staff training and development strategy were significant predictors of hospital performance [19]. Also, in the study by Samara (2014), recruitment strategy, staff training and development strategy, and motivation strategy had the greatest impact on the performance of Jordanian public hospitals [6].

The discrepancy in some study results may be due to the hospital management focusing more on some human resource strategies, which has led to the same strategies having a greater impact on the overall performance of the hospital. Of course, it should be borne in mind that the type of employees' perceptions of human resource strategies is also important. In short, we can say that most of these findings are consistent with this study. It seems that hiring qualified medical staff and their training and career development have a significant positive effect on hospital performance. By applying appropriate human resources strategies, individual productivity and organizational performance can be expected to be higher.

The findings of this study showed that the impact of HRS on OP in non-financial perspectives (Including; learning & growth, customer and internal business process) was greater than the financial perspective. In this regard, Vermeeren et al. (2016), who conducted a study in 162 Dutch healthcare institutions, showed that human resources management activities and strategies have a positive effect on financial (Measure: net margin), organizational (Measure: client satisfaction), and human resources outcomes (Measure: sickness absence). But in the meantime, the impact of human resources activities on non-financial outcomes was much greater than the financial outcomes [2]. In addition, Valmohammadi and Ahmadi (2015) found that knowledge management is only effective on OP in the learning and growth perspective [14]. These studies support our findings. It seems HRS affect the organizational performance in non-financial dimensions more than financial performance.

While most related studies have investigated the impact of HRS on overall hospital performance, Michael et al. (2006) examined the impact of policies and practices of human resources management on standardized patient mortality rates in 52 UK hospitals. Their study showed that these policies and practices had a significant effect on reducing patient mortality [20].

Human resources are key factors in gaining an organization's competitive advantage. Therefore, to achieve the optimal OP, it is necessary to effectively manage human resources through appropriate strategies, in such a way that these strategies are consistent with the strategy of the organization [21]. When human resources systems focus on strategic decisions instead of emphasizing individual actions, it will have a significant impact on OP [25]. Since the attitude of healthcare workers is an important element of hospital performance [2], the development of research in this area can provide useful information for improving the performance of these institutions.

### Limitations of the study

Research in one institution, the type of study (descriptive & cross-sectional), small statistical population, low participation of physicians in the study, and self-reported method in completing the questionnaire are the limitations of this study, which can affect the generalizability of the findings.

## Conclusion

This study showed that there was a strong positive relationship between HRS and OP. Based on our findings, HRS affected the OP in non-financial dimensions more than financial performance. In addition, training and development strategy, staff compensation & reward strategy, and recruitment strategy had the strongest relationship with OP respectively. To improve hospital performance, the development of HRS (especially training and development, compensation, and recruitment strategies) can be used by hospital managers.

Measuring the performance of the organization ensures the continuity of the organization's competitiveness in the global market. On the other hand, performance indicators are typically achieved through human resources. Therefore, to achieve optimal organizational performance, it is necessary to effectively manage human resources through the development and application of human resources strategies.

Although this study is a small-scale research, the findings could be applied for other public hospitals in Iran and other hospitals that have similar conditions. Our study contributes to the development of research in this field.

## Data Availability

The datasets used in this study are accessible by the corresponding author on the reasonable request.

## Abbreviations

HRS: Human Resources Strategies
OP: Organizational Performance
BSC: Balanced ScoreCard
